# Parosmia COVID-19 Related Treated by a Combination of Olfactory Training and Ultramicronized PEA-LUT: A Prospective Randomized Controlled Trial

**DOI:** 10.3390/biomedicines11041109

**Published:** 2023-04-06

**Authors:** Arianna Di Stadio, Elena Cantone, Pietro De Luca, Claudio Di Nola, Eva A. Massimilla, Giovanni Motta, Ignazio La Mantia, Gaetano Motta

**Affiliations:** 1 Department GF Ingrassia, Otolaryngology Unit, University of Catania, 95131 Catania, Italy; 2 Department of Otolaryngology, Federico II University, 80131 Naples, Italy; 3 Department of Otolaryngology, San-Giovanni Addolorata Hospital, 00100 Rome, Italy; 4 Department of Otolaryngology, Volvatellid University, 81055 Naples, Italy

**Keywords:** smell loss, parosmia, qualitative smell disorders, treatment, PEA, olfactory training

## Abstract

During COVID-19 pandemic, clinicians have had to deal with an ever-increasing number of cases of olfactory disturbances after SARS-CoV-2 infections and in some people this problem persisted for long time after negativization from virus. This a prospective randomized controlled trial aims at evaluating the efficacy of ultramicronized palmitoylethanolamide (PEA) and Luteolin (LUT) (umPEA-LUT) and olfactory training (OT) compared to OT alone for the treatment of smell disorders in Italian post-COVID population. We included patients with smell loss and parosmia who were randomized and assigned to Group 1 (intervention group; daily treatment with umPEA-LUT oral supplement and OT) or Group 2 (control group; daily treatment with placebo and OT). All subjects were treated for 90 consecutive days. The Sniffin’ Sticks identification test was used to assess the olfactory functions at the baseline (T0) and the end of the treatment (T1). Patients were queried regarding any perception of altered olfaction (parosmia) or aversive smell, such as cacosmia, gasoline-type smell, or otherwise at the same observational points. This study confirmed the efficacy of combination of umPEA-LUT and olfactory training as treatment of quantitative smell alteration COVID-19 related, but the efficacy of the supplement for parosmia was limited. UmpEA-LUT is useful for the treatment of brain neuro-inflammation (origin of quantity smell disorders) but has limited/no effect on peripheral damage (olfactory nerve, neuro-epithelium) that is responsible of quality disorders.

## 1. Introduction

Smell disorders are classically divided into two main categories, they can be quantitative and qualitative. Hyposmia and anosmia are quantitative disorders diagnosed and managed more easily than qualitative disorders namely parosmia and phantosmia, that are areas of research more open to interpretation. Unlike phantosmia, in which distorted smell detection occurs without any smell stimuli to trigger it, parosmia is a distortion of smell detection in the presence of smell stimuli. The two subtypes of parosmia are cacosmia, the detection of unpleasant smell and euosmia the detection of pleasant smell. 

Although parosmia was an already known condition but with limited clinical interventions, it is only after the COVID pandemic that researchers have had to deal with an ever-increasing number of cases, thus contributing to an accumulation of knowledge and new approaches [1]. 

For instance, about 35% of people suffering from smell disorders are found to have parosmia, but the prevalence of smell disorders in COVID-19 infections are up to 75% and up to 45% for parosmia [2,3,4,5].

Generally, parosmia recovers in the first 1–2 months after the onset of symptoms but recent studies emphasize an improvement in parosmia even after 40 years of symptoms. [6]

The highly variable durations of parosmia explain why qualitative smell disorders require multiple approaches and treatments. This significant variation must induce clinicians to approach a therapeutic protocol even in cases of long-lasting parosmia. In addition, some patients could take up to 6 months to recover, so it is important to extend treatment duration [7,8].

Recent literature demonstrated that traditional treatment methods as olfactory training and steroids were rarely effective in the treatment of post-COVID parosmia highlighting the importance of finding new therapeutic approaches [9].

Because of the origin of parosmia and the absence of a treatment we speculated that a treatment by an anti-neuroinflammatory molecule could benefit. Di Stadio in 2022 [5] identified a high prevalence of parosmia in their patients affected by smell disorders COVID-19 related; the authors justified the presence of this symptom because of neuro-inflammatory process in the olfactory bulbs [10,11]. The inflammation, in the authors opinion, negative impact on the recovery causing the persistence of the qualitative disorder [12].

Several authors showed the efficacy of umPEA-LUT to fight the inflammation caused by COVID-19 infection [13,14,15], as well as the ability of the molecule of inhibiting the penetration of the SARS-CoV-2 virus protecting people from the infection [16].

Different studies have shown the efficacy of combination between a supplement containing ultramicronized-palmytoiletanolamide and luteolin (umPEA-LUT) and olfactory training in ameliorating the quantitative smell alteration in patients who suffered from this persistent concern [17,18,19]. 

This randomized clinical trial aims at evaluating the efficacy of umPEA-LUT on the quality smell disorders in Italian post-COVID population suffering from parosmia following daily treatment with PEA-LUT and olfactory training compared to conventional therapy. 

## 2. Materials and Methods

### 2.1. Study Population and Demographic Data

This prospective randomized controlled trial was conducted in a tertiary referral hospital of Naples from January 2022 to December 2022. A total of 130 people were included in this trial. The randomized controlled trials registration number is NCT04853836.

For patients’ recruitment, we used both clinician communication and mass media. At the moment of recruitment, we assigned a number to the patient, and we elucidated that the study aim was to identify new methods for treating persistent loss of smell after COVID-19. We informed patients that after baseline assessment they would be re-checked after 90 days, with up to two possible intermediary olfactory assessments, as dictated by protocol. Patients were aware that their participation was voluntary and that they could leave the study at any time. To guarantee the blinding we divided the investigations in two; one physician, who did not know the assignation to the experimental group, performed the endoscopy to study the baseline condition (e.g., polyps or tumors), and another did olfactory testing using validated measures of threshold, discrimination, and identification scores. Self-report data on mental clouding/brain fog was also collected. The anonymized data were recorded on a protected Excel sheet [Google (Mountain View, CA, USA)].

Study participants were included or excluded based on the following criteria.

#### 2.1.1. Inclusion Criteria

Inclusion criteria for outpatients were ages between 18 and 65 years, confirmed history of COVID-19 by positive nasopharyngeal swab for SARS-CoV-2), and anosmia/hyposmia identified by using the 16-pen version of the Sniffin’ sticks psychophysical test (I score 0–16), olfactory impairment persisting ≥180 days (6 months) after sub-sequent negative COVID-19 nasopharyngeal swab, acceptance to participate to the study by signature of written consent.

#### 2.1.2. Exclusion Criteria

We identified specific exclusion criteria like history of smell and taste alterations, current or past alterations of memory or cognitive functions, current under chemotherapy or estaromatase, neuroinflammatory/neurodegenerative diseases in active phase, use of therapy able to alter smell and taste, active rhino-sinusitis including allergic and athropic rhinitis, previous chemo-radiotherapy in the head/neck, previous removal of tumor in nose or paranasal sinuses, recent history (<3 years) of stroke or moderate/severe head trauma, nasal septal deviation or turbinate hypertrophy, recent use of steroid nasal therapy within 30 days from the enrollment. The patients who were using drugs that could either independently reduce inflammation or interfere with PEA-LUT were excluded from the study.

### 2.2. Demographic Data Extraction

All this demographic information was collected for each patient included in the study: age, sex, time elapsed since negative COVID-19 test, prior treatment for olfactory disorders, presence of major disease, tobacco/alcohol use and medications. We designed a personal medical record to collect info about current and history of systemic diseases, including previous olfactory disorders or neuroinflammatory/neurodegenerative concerns, details about COVID as onset symptoms, swab results, treatments used, symptom persistence and COVID vaccine. The medical recorder also contained a section to annotate patient’s self-perception of the smell alteration; we explained the differences and type of quantitative and qualitative smell alteration before asking for this specific info. After three from the beginning of the therapy the data of the patients who concluded the therapeutic scheme were extracted and analyzed by a statistician supervised by the study coordinator (ADS).

### 2.3. Experimental Groups

The consecutive patients enrolled in the study were assigned to 2 groups, as follows:

1. Intervention therapy (intervention group): Daily treatment with co-ultra-micronized PEA 700 mg and Luteolin 70 mg (umPEA-LUT) (Glialia ^®^, Epitech Group SpA, Milano, Italy) ultraPEA-LUT oral supplement and olfactory training. The oral supplement was prescribed as single dose to be assumed 5–10 min before breakfast in combination with daily olfactory training. The latter was done using four 100% organic essences of Lemon, Rose, Eucalyptus and Cloves three times a for 6 min each session; the olfactory stimulus consisted in smelling an odor for 4–6 s, then 40 s of relaxation, and then, new stimulation for 4–6 s with another odor. The short duration-stimulus was necessary to avoid “saturation” of the olfactory receptors [20]. This treatment was performed for 90 consecutive days.
2. Conventional therapy (control group): patients assigned to this group performed daily olfactory training exactly as previously described plus a placebo supplement therapy (multivitamin, vitamin D (400 UI), and/or alpha-lipoic acid (120 mg). The dosages used for vitamin D and of alpha-lipoic acid were selected based on an evidence-based literature review that showed these dosages did not have significant systemic anti-inflammatory, immunomodulatory, or antioxidant effects [13,14,15,18,19].

For ethical reason it was not possible to recruit patients and leave them without any treatment, creating in this way a “real” control group. By the way, because we included patients who were without treatment for at least 6 months; this could be supportive of the concept that without any treatment, neither OT nor supplement, there was no recovery and could justify this study design that compared traditional therapy (OT performed in control) and OT plus umPEA-LUT.

All patients performed their house-olfactory training (self-administered rehabilitation) after being adequately trained by the physician. Initially, the patients were trained in the hospital by face-to-face explanation on how to perform the sniffing exercise, practice performing the exercise. A written description on how to prepare the sniffing essence was given to each participants and for detailed instruction we provided a “YouTube” link (https://www.youtube.com/watch?v=Ri5YwM6EmWM; accessed on 1 January 2022). The video used was previously selected by the study coordinator and it was always the same for all patients.

To follow our participants and promote adherence to the study, members of clinic staff and physician regularly contacted people (1 contact each 15 days) by via phone calls, electronic communications, and office visits.

### 2.4. Nasal Endoscopy Assessment of Olfactory Dysfunction

*A* nasal endoscopy to exclude nasal conditions was always performed to identify conditions that could alter the olfactory functions or interfere with the treatment. In presence of conditions described in the exclusion criteria (for example nasal mass prior impaired smell, history of nasal/nasopharyngeal malignancy, history of radiation, or other anatomical abnormalities) the patients were excluded.

### 2.5. Assessment of Olfactory Dysfunction

The Sniffin’ Sticks identification test was administered to assess olfactory function following a previously established protocol [16]. Briefly, clinicians used standard pen-like devices filled with odorants to score olfactory function and were blinded to the patients’ experimental groups. During the odor identification task, participants were presented with 16 common odors. In multiple-choice format, participants were asked to select which of 4 odor labels matched the presented odor. Possible scores for odor identification ranged from 1–16. These scores were used classify olfactory function as anosmia (score <7), hyposmia (score from 7–14), or normosmia (score ≥14), and scores were then recorded for subsequent analysis.

### 2.6. Parosmia Evaluation

Patients after being educated about the quality alterations of the smell were asked about presence of parosmia (distorted perception of smell) or cacosmia (aversive smell like gasoline-type odor). This method, which modified the questionnaire used by Di Stadio et al. [11], was validate by De Luca et al. [19] on 100 patients.

### 2.7. Statistical Analysis

One-way ANOVA for repeated measures and Bonferroni-Holmes (BH) post-hoc tests were used to compare the results of the Sniffin’ stick score at T0 and T1 between treatment and control groups. Chi-square (c) was used to compare nominal data represented by the changes or not of the parosmia after treatment considering both groups, and to evaluate the recovery based on the >3 points, <3 points, stable or worsening. Multi-linear regression analyses were performed to evaluate the effect of age, sex and TDI at T0 (x variables) on TDI at T1 (y variables).

Multi-linear regression including age, sex, smoke, TDI at T0 and TDI at T1 (x variables) and parosmia (y variable) were performed in each group to identify which of the parameters could affect the presence/absence of parosmia. *p* was considered significant < 0.05.

## 3. Results

A total of 130 patients were included in the study. Because the efficacy of combination between umPeaLut and olfactory training was known [12,13,14,15], the allocation of patients in the treatment was 4:2.7.

94 patients (49 women and 45 men, age average 36.7 ± 11.8) were assigned to the treatment group and 36 (21 women and 15 men, age average 50.5 ± 12.7) to the control group.

The patients in treatment group suffered from smell alteration for 8.8 ± 3.7 months average, patients in the control for 8.5 ± 2 months average.

Table 1 shows the demographic characteristics of the two groups including comorbidities and previous treatments performed for smell loss.

Most of the patients both in treatment (83) and in the control (33) did not perform brain MRI, however the ones who did, 11 in treatment and 6 in the control group did not show olfactory bulbs atrophy.

14 patients (14.9%) in the treatment group were smokers, 9 (27.2%) in control.

At the baseline (T0) 48 patients were anosmic and 46 hyposmic in the treatment group, while in the control group 12 were anosmic and 24 hyposmic.

The average Sniffin’ score at the baseline (T0) was 8.2 ± 2.7 (CI95%: 2–15) in the patients assigned to the treatment group and 9.6 ± 2.4 (CI95%: 8–13) in the control. The difference between the two groups at the T0 was not statistically significant (*p* > 0.05).

The average Sniffin’ score at T1 was 11.1 ± 2.2 in the treatment group and 10.1 ± 2.3 in the control.

In the treatment group, despite sex, age and TDI at T0 influenced the Sniffin’ score at T1 (*p* < 0.00001), the only variable that had statistically significant impact on the final results was the Sniffing score at T0 (*p* < 0.00001).

In the control, despite sex, age and TDI at T0 affected the Sniffin’ score at T1 (*p* = 0.0009), as well as observed in the treatment group, the only variable that had statistically significant impact on the Sniffin’ score at T1 was the Sniffing score at T0 (*p* = 0.009).

**Figure 1:**
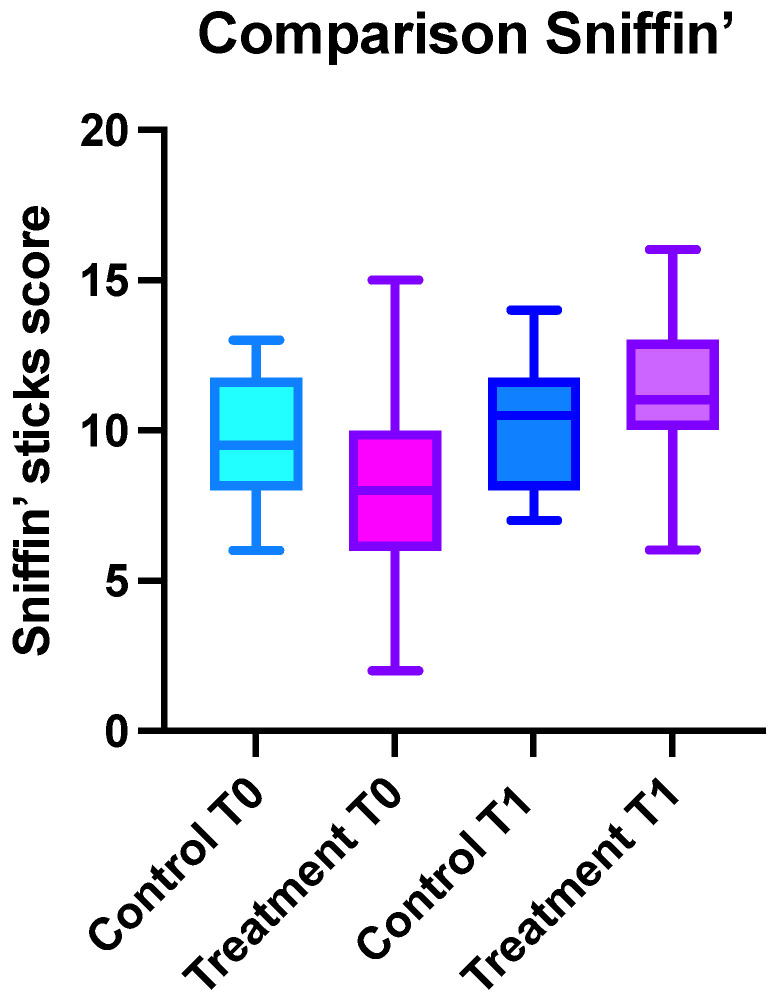
Comparison between the Sniffin’ Score at T0 and T1 both in the treatment group and in control group. A statistically significant difference was observed T0 and T1 in the treatment group (ANOVA: *p* < 0.0001; BH: *p* < 0.01), and between treatment and control group at T1 (HB: *p* < 0.05) but not statistically significant difference comparing the Sniffin’ score at T0 and T1 in the control (HB: *p* > 0.05).

We identified statistically significant differences comparing T0 and T1 in the treatment group (ANOVA: *p* < 0.0001; BH: *p* < 0.01), but not statistically significant difference comparing the Sniffin’ score at T0 and T1 in the control (HB: *p* > 0.05). Statistically significant differences were observed between treatment and control (HB: *p* < 0.05) at T1(Figure 1).

**Figure 2:**
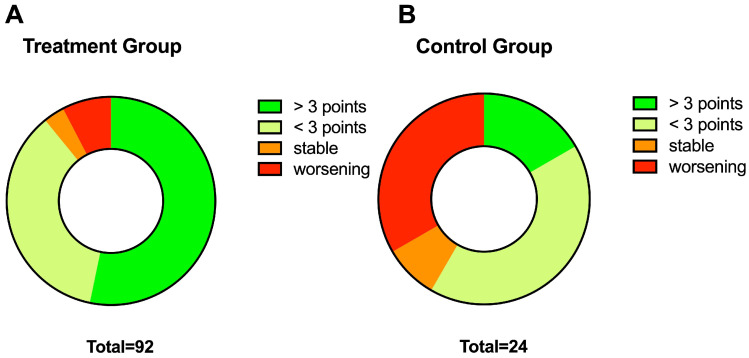
This figure shows the comparative results in term of recovery between the treatment group and the control group. (**A**) the graph shows the results obtained in the treatment group; (**B**) the figure illustrates the results in the control group.

49 patients (52.1%) in the treatment (average 11.4 ± 2.5, CI95%: 8–16) and 6 (18.2%) in the control (average 10.5 ± 0.7; CI95% 10–11) recovered more than 3 points at T1. 33 (35.1%) in the treatment (average 10.6 ± 1.7, CI95%: 8–13) and 15 (45.4%) (average 11.2 ± 2.4, CI95%: 8–14) in the control recovered less than 3 points. 3 patients (3.2%) in the treatment group did not change they score comparing T0 and T1 (average 12.6 ± 0.6, CI95%: 12–13), while in the control group three subjects (9.1%). Finally, we observed 7 patients (7.4%) in the treatment group that worsened their Sniffin’ score comparing T0 and T1 (average 10.6 ± 2.5, CI95%: 6–14) and 12 (36.4%) in the control presented the same concern (average 8.5 ± 2.4, CI95%: 7–12). The differences between treatment and control in term of recovery were statistically significant (c: *p* = 0.0008) (Figure 2A,B).

**Figure 3:**
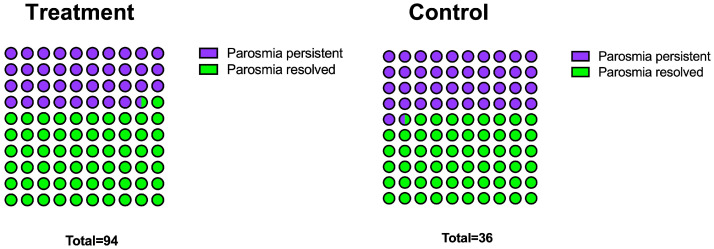
Comparison between the patients who recovered from parosmia in treatment group and in control group; the difference was not statistically significant (χ: *p* = 0.3).

The totality of the patients in both groups had associated parosmia before the treatment. After treatment 58 patients (61.7%) in the treatment group and 21 (58.3%) in the control resolved the parosmia. The difference was not statistically significant (χ: *p* = 0.3) between treatment and control at T1 (Figure 3).

The multilinear regression analysis showed that in the treatment group the score of the Sniffin’ score at T1 were not correlated to the resolution of the parosmia (*p* = 0.06), while in the control this was related to the age (*p* = 0.0007) and the Sniffin’score at T0 (*p* = 0.02).

## 4. Discussion

This prospective randomized controlled trial confirmed the efficacy of combination between olfactory training and umPEA-LUT as treatment of quantitative post COVID smell disorders; the efficacy of the treatment on qualitative smell disorders should be deeply investigated because several co-factors other than the Sniffn’scores can impact on the persistence or resolution of parosmia after treatment.

Based on the hypothesis that quantitative smell disorders could be related to the inflammation in the olfactory bulbs, while qualitative disorders might be caused by the damage of neuro-epithelium [5] the absence of statistically significant difference regarding the recovery from parosmia that we observed was totally predictable. On the other hand, Di Stadio et al. speculated that the use of umPEA-LUT might help even in case of parosmia because the qualitative disorders might be also caused by an abnormal regeneration of the olfactory nerve; our study, despite preliminary because performed on a small sample of patients, seems confirming the hypothesis that neuro-epithelium is responsible of parosmia. UmPEA-LUT is a drug active on the central structures (olfactory bulbs) rather than on the peripheral ones (olfactory nerve and neuro-epithelium) [18] and this can explain why, despite an important effect on the recovery of the smell, the compound did not do the difference on the quality of odors perception between treatment and control groups.

In this study, patients in the treatment group started with worsen quantitative smell problems than control (respectively 8.2 ± 2.7 and 9.6 ± 2.4); however, despite that, the patients in the treatment group recovered as well as the ones in the control, but with more homogeneous scores (lower standard deviation than control). All subjects included were affected by olfactory disorders from 8.5 months at least (8.8 treatment and 8.5 control) so part of the recovery might be spontaneous; in fact, some authors reported recovery of olfactory functions by spontaneous recovery within two years [21] but the authors did not reported info about the SS. Moreover, in this study over 60% of patients did not recover fully olfactory capacities. Probably the concept of spontaneous recovery should be re-evaluated even considering that the treatment with umPEA-LUT and olfactory training allowed the return to normal olfactory function in over 60% of patients as previously showed [18].

This current study confirms the efficacy of the Di Stadio et al. protocol [14,15]; 80% patients treated with umPEA-LUT improved their SS in most of cases with gain over 3 points. On the contrary, in control less than 25% of patients recovered over 3 points and the likelihood of worsening was around 30%.

We noted that the only variable that affected the SS at T1 both in the control and treatment group with statistically significant impact was the score that patients had at the baseline (T0); this result confirms previous studies that did not identify an effect of the age and gender on the recovery of the olfactory function in young population (<60 ies) [2,3,18,20].

Anyway, although only SS at T1 was correlated (not statistically significant *p*) with the resolution of parosmia, no statistically significant differences for this finding were observed between treatment and control. In the latter the resolution of the qualitative disorder was correlated (with statistically significant *p*) with age and SS at T0. Because patients in control were younger than treatment and McWilliams et al. showed that in patients <40 spontaneously recovery of smell is likely [21], the improvement of parosmia in the control could be related to the young age rather than to the olfactory training. Additional study with the same design but with homogeneous ages among groups should be performed to understand if umPEA-LUT could really benefit the qualitative smell problems [21].

Although only few patients performed brain MRI both in the treatment and control group, in none of them we identified an atrophy of the olfactory bulbs. The atrophy of these structures was identified as negative prognostic factors in the recovery of normal olfactory functions [22]. Other authors showed that a change in the shape of the olfactory bulbs [23], without atrophy, could be observed in patients with post-COVID anosmia. The authors speculated that these changes could be related to the neuro-inflammation [23] and this could explain why, despite absence of atrophy, the patients in our sample suffered from anosmia. The use of umPEA-LUT, which reduces neuro-inflammation [13,14,15,18,19], improved the quantitative smell functions in our patients; additional study using 3T MRI and/or functional MRI focused on the olfactory bulbs could elucidate the mechanism of action of the supplement.

Overall, this study confirmed the efficacy of the combination of umPEA-LUT and olfactory training to recover quantitative olfactory functions, but additional study with age and sex stratification should be performed to understand the benefit of this treatment for qualitative disorders.

Our group is currently testing a new combination of supplements (umPEA-LUT and alpha-lipoic acid) to solve both quantitative and qualitative smell problems.

### Limitations of the Study

The present study has some limitations. First, we only did odor identification, and the lack of a comprehensive battery of olfactory assessments incorporating thresholds and discrimination (complete TDI score) it is extremely important especially in patients with parosmia. Second, we used a previously published method for measuring parosmia, but this tool is not well-validated due to the limited published experience with COVID-19 parosmia, and it relies on subjective patient assessments. Third, the number of patients quite differed between the treatment group and the control group, and this disproportion could affect our results. Last, there is a significant difference between the mean age of the two groups, and this could be another confounding factor.

Despite these limitations, the prospective nature of the study, the presence of control group, the strengthens of the statistical analysis, and the promising results are the strengths of the present analysis.

## 5. Conclusions

This study confirmed the efficacy of combination of umPEA-LUT and olfactory training as treatment of quantitative smell alteration COVID-19 related. The results we obtained regarding the efficacy of the same treatment on parosmia are limited and their interpretation has been impacted by the difference in age average among the groups. In fact, control group included younger patients than treatment, who could have benefit by spontaneous recovery which is less likely in patients over 40 ies; this finding was confirmed by the identification of age and sniffing score at T0 as elements that impacted on the parosmia resolution. Additional studies comparing patients with same age are necessary to understand if umPEA-LUT might be helpful for the treatment of qualitative smell alterations.

Moreover, we are currently evaluating a combination between umPEA-LUT and alpha lipoic acid to understand if this combination of supplements associated with olfactory training can help to recovery full sense of smell.

## Figures and Tables

**Table 1:** Characteristic of patients of patients included in the study.

Variable	Group 1	Group 2
	(*n* = 94)	(*n* = 36)
**Mean age ± SD (range), yr**	36.7 ± 11.8	50.5 ± 12.7
**Gender—no (%)**		
Female	49 (52%)	21 (58%)
Male	45 (48%)	15 (42%)
**Smoking habits**		
Smokers	14 (15%)	6 (16.6%)
Non-smokers	80 (85%)	18 (50%)
**Comorbidities**		
Hypertension	1 (1%)	2 (5%)
Thyroid Disorders	6 (6%)	2 (5.5%)
Allergy	5 (5%)	2 (5.5%)
Psychiatric disturbances	2 (2%)	6 (16%)
**Time from symptoms onset (mean ± SD, range), months**	8.8 ± 3.68 (3–15)	8.5 ± 1.97 (5–12)
**Type of olfactory disfunction—no. (%)**		
Anosmia	39 (41.5%)	8 (22%)
>Hyposmia alone	0 (0%)	14 (39%)
Parosmia/Cacosmia alone	7 (7.5%)	2 (5.5%)
Hyposmia ± Parosmia/Cacosmia	48 (51%)	14 (39%)
**T0 Identification score (Sniffin’ Sniff Test) (mean ± SD, range)**	8.2 ± 2.7	9.6 ± 2.4
**T1 Identification score (Sniffin’ Sniff Test) (mean ± SD, range)**	11.1 ± 2.2	10.1 ± 2.3

SD—standard deviation.

## Data Availability

Data are available under request to the corresponding author.
